# Reactive hyperemia correlates with the presence of sepsis and glycocalyx degradation in the intensive care unit: a prospective cohort study

**DOI:** 10.5935/0103-507X.20200064

**Published:** 2020

**Authors:** Luís Filipe Gomes Malheiro, Rita Gaio, Manuel Vaz da Silva, Sandra Martins, Susana Sampaio, Janete Quelhas-Santos, Ana Cerqueira, António Sarmento, Lurdes Santos

**Affiliations:** 1 Department of Infectious Diseases, Centro Hospitalar de São João, Faculdade de Medicina, Universidade do Porto - Porto, Portugal.; 2 Department of Mathematics, Faculdade de Ciências, Universidade do Porto - Porto, Portugal.; 3 Department of Pharmacology & Therapeutics, Faculdade de Medicina, Universidade do Porto - Porto, Portugal.; 4 Department of Clinical Pathology, Centro Hospitalar de São João, Faculdade de Medicina, Universidade do Porto - Porto, Portugal.; 5 Department of Nephrology, Centro Hospitalar de São João, Faculdade de Medicina, Universidade do Porto - Porto, Portugal.

**Keywords:** Hyperemia, Sepsis, Endothelial dysfunction, Syndecan-1, Peripheral arterial tonometry, E-selectin, Endothelium, vascular/physiopathology, Glycocalyx, Hiperemia, Sepse, Disfunção endotelial, Syndecan-1, Tonometria arterial periférica, E-selectina, Endotélio vascular/fisiopatologia, Glicocálix

## Abstract

**Objective:**

To investigate whether reactive hyperemia measured by peripheral arterial tonometry correlates with markers of endothelial dysfunction and may be used to identify sepsis in critical illness.

**Methods:**

A prospective study was performed using a cohort of critically ill patients. Endothelial dysfunction was assessed on admission by quantifying reactive hyperemia-peripheral arterial tonometry and plasma levels of endothelin-1, soluble E-selectin, endocan and syndecan-1. Septic patients were compared to patients without evidence of infection.

**Results:**

Fifty-eight septic patients were compared to 28 controls. The natural logarithm of reactive hyperemia-peripheral arterial tonometry was negatively correlated with cardiovascular comorbidities, disease severity and plasma levels of soluble E-selectin (p = 0.024) and syndecan-1 (p < 0.001). The natural logarithm of reactive hyperemia-peripheral arterial tonometry was lower in septic patients than in controls (0.53 ± 0.48 versus 0.69 ± 0.42, respectively). When adjusted for age, the multivariable model predicted that each 0.1-unit decrease in natural logarithm of reactive hyperemia-peripheral arterial tonometry increased the odds for infection by 14.6%. m.

**Conclusion:**

Reactive hyperemia-peripheral arterial tonometry is closely related to soluble E-selectin and syndecan-1, suggesting an association between endothelial activation, glycocalyx degradation and vascular reactivity. Reactive hyperemia-peripheral arterial tonometry appears to be compromised in critically ill patients, especially those with sepsis.

## Abstract (pt)

**Objetivo:**

Investigar se a hiperemia reativa correlaciona-se com marcadores de disfunção endotelial e pode ser utilizada para identificar sepse na doença crítica.

**Métodos:**

Trata-se de estudo prospectivo em uma coorte de pacientes críticos. A disfunção endotelial foi avaliada quando da admissão, por meio da quantificação de hiperemia por tonometria arterial periférica e níveis plasmáticos de endotelina 1, E-selectina solúvel, endocana e sindecano 1. Os pacientes sépticos foram comparados com pacientes sem evidência de infecção.

**Resultados:**

Cinquenta e oito pacientes sépticos foram comparados com 28 controle. O logaritmo natural da tonometria arterial periférica teve correlação negativa com comorbidades cardiovasculares, severidade da doença e níveis plasmáticos de E-selectina solúvel (p = 0,024) e sindecano 1 (p < 0,001). O logaritmo natural da tonometria arterial periférica foi mais baixo nos pacientes sépticos quando comparado com os de pacientes controle (0,53 ± 0,48 versus 0,69 ± 0,42, respectivamente) e, quando ajustado à idade, o modelo multivariado predisse que cada 0,1 de diminuição em unidades de logaritmo natural da tonometria arterial periférica levou a aumento de 14,6% na probabilidade de infecção.

**Conclusão:**

A hiperemia reativa avaliada por tonometria arterial periférica tem estreita relação com E-selectina solúvel e sindecano 1, o que sugere associação entre ativação endotelial, degradação de glicocálix e reatividade vascular. A hiperemia reativa por tonometria arterial periférica parece estar comprometida em pacientes críticos, especialmente os com sepse.

## INTRODUCTION

Diagnosing sepsis is challenging. It requires the often unclear identification of infection alongside the identification of sepsis-induced organic dysfunction.^(1)^ Mortality correlates with the severity of sepsis and may exceed 40% when septic shock is present, highlighting the importance of prompt recognition of sepsis and adequate treatment.^(2)^ However, there is no gold standard in the diagnosis of sepsis. Current recommendations suggest using the Sequential Organ Failure Assessment (SOFA) score; however, it does not reflect the full extent of the sepsis mechanisms, and several authors have criticized its lack of specificity along with other limitations.^(2-7)^

Sepsis is an intricate network of immunologic responses, and many targets have been investigated in the hope of identifying a dynamic biomarker that would help distinguish septic from nonseptic patients. Unfortunately, many of these biomarkers are restricted to research or are expensive or nonspecific.^(8-14)^

Sepsis-induced acute endothelial dysfunction is a consequence of circulating cytokines and bacterial and viral toxins and has protean manifestations that include microvascular dysfunction, coagulation disorders, increased vascular permeability and loss of vascular tone.^(15-18)^ All these changes cause perfusion defects and cell death, triggering organ damage.^(8)^ Reactive hyperemia (RH) is a transient nitric oxide (NO)-dependent vasodilatory response that occurs briefly after a period of flow occlusion, is part of the normal microvascular response to volume changes, and is highly dependent on the endothelial glycocalyx integrity.^(19-23)^ Peripheral arterial tonometry (PAT) is a noninvasive and indirect method of evaluating the RH response (RH-PAT) with adequate reproducibility and is associated with chronic and acute endothelial dysfunction.^(21,24-27)^

Sepsis-induced damage to the endothelial glycocalyx and NO-depletion may contribute to endothelial dysfunction and microvascular dysfunction.^(17,18)^ Reactive hyperemia-peripheral arterial tonometry is impaired in septic patients compared with healthy controls, but the specificity of this impairment to sepsis compared with other critically ill patients with noninfectious disease remains unknown.^(23,28-30)^

The objective of this study is to evaluate whether RH-PAT correlates with the diagnosis of sepsis and immunologic markers of endothelial activation and whether it can be used to identify sepsis in critically ill patients in clinical practice.

## METHODS

This was a prospective study of a cohort of consecutive patients admitted to the intensive care unit (ICU) of the Infectious Diseases Department in a single tertiary care center. This study was approved by the local ethics committee (*Centro Hospitalar de São João*, ref. 205/11) and was performed in accordance with the ethical standards of the 1964 Declaration of Helsinki and its later amendments. Written consent was obtained from the patient if awake or from the legal representative.

Consecutive adult patients admitted to the ICU over a period of 12 months were evaluated for eligibility in the first 24 hours following admission. Patients were stabilized according to routine care in the ICU. The study protocol did not interfere with daily activity. Patients were considered eligible if they fulfilled the following criteria: presence of a disorder (infectious or not) with a severity that justifies admittance to the ICU for surveillance or therapy. Exclusion criteria were unavailability to perform RH-PAT in the first 24 hours or clinical and anatomic conditions that interfere with PAT, such as absence of the 2nd or 3rd pair of fingers, severe hypotension (such that compression of the brachial artery would compromise distal perfusion), brachial artery stenosis or severe reactive vasospasm in response to recent arterial catheterization, arterial-venous fistulae in any arm, permanent tremor or restlessness and platelets < 20.000/µL or coagulation disorders.

Patients were classified as septic according to the SEPSIS-3 definition.^(2)^ The patient was considered infected with a confirmed microbiologic etiology on appropriate diagnostics (culture, blood smear, serology, antigen detection or nucleic acid amplification test) or by clinical response to antimicrobial therapy in the absence of microbiological identification where an alternative diagnosis was not likely. Controls were defined when there was no evidence of an infection on admission or no need for antimicrobials and when an alternative diagnosis was made. Patients were further subclassified into septic shock, cardiogenic shock or hypovolemic shock categories according to current guidelines.^(2,31)^

On the first 24 hours of admittance to the ICU, each included participant underwent clinical and demographic data collection, routine blood sampling for biochemical analysis and biomarkers quantification and RH-PAT evaluation. For each patient, plasma was aliquoted and stored at -80ºC for further biomarker quantification. To simplify the multivariable analysis, age was categorized with a cut-off value of 60 years old because this was the most reasonable cut-off after which endothelial dysfunction is independently and significantly affected by age according to studies evaluating the effect of age on endothelial function.^(32)^ For each participant, the SOFA, Acute Physiology and Chronic Health Evaluation II (APACHEII) score and Simplified Acute Physiology Score II (SAPSII) were quantified.

### Reactive hyperemia measured by peripheral arterial tonometry quantification

Endothelial dysfunction was measured indirectly by quantification of RH-PAT using the Endo-PAT 2000® tonometer (Itamar Medical, Cesarea, Israel). The evaluation was performed after at least 1 hour of hemodynamic stability (defined as absence of vasopressor drugs dose adjustments or need for fluid bolus).

Assessments were performed in a controlled environment (room temperature 21.5ºC; humidity 48.9%) with minimal distractions in the dorsal recumbent position. When present, vasopressors doses were stable for ≥ 60 minutes, and only essential routine care was performed during the evaluation. For each assessment, a software profile was introduced, controlling for age, weight, estimated height, heart rate and arterial pressure.

**Figure 1:**
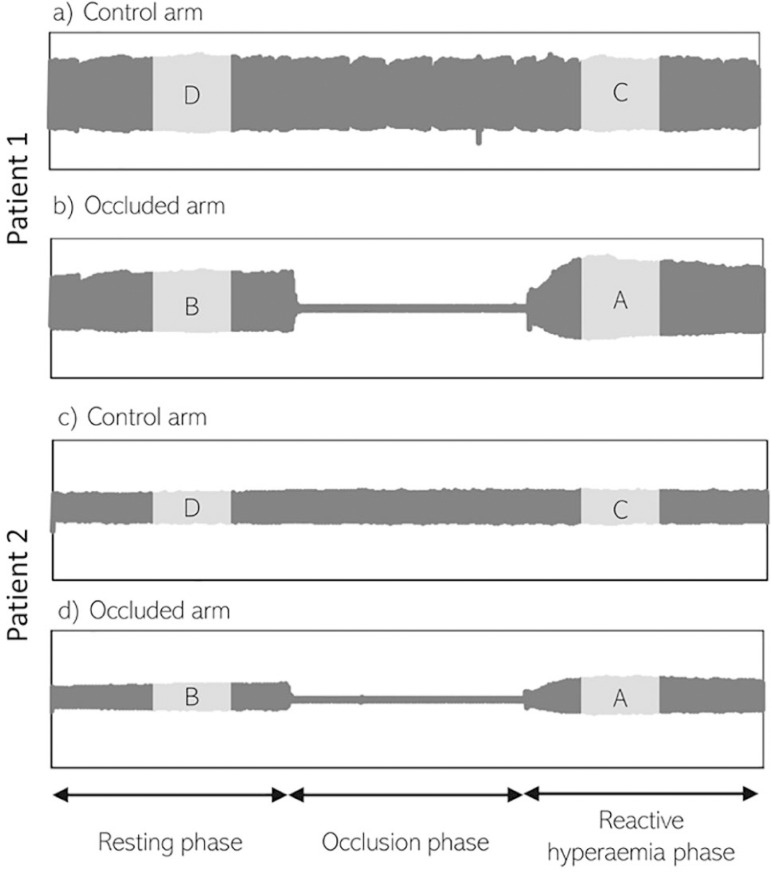
Reactive hyperemia evaluation by peripheral arterial tonometry in two different patients. (A and B) A patient with a normal reactive hyperemia index. (C and D) A patient with an impaired reactive hyperemia index. A finger plethysmograph captures pulse wave amplitude after each cardiac beat. Following a baseline evaluation for 5 minutes in a resting state, a cuff is inflated in one of the arms for 5 minutes. After deflation, pulse wave amplitude is registered for another 5 minutes, and the mean pulse wave amplitude is calculated for the interval of 90-150 seconds postocclusion. The reactive hyperemia index is reported as the ratio of the occluded arm’s mean pulse wave amplitude post occlusion (A) to the mean pulse wave amplitude from baseline readings of the same arm (B), and the result is further divided by the same ratio in the control arm (C/D), allowing the device to account for systemic vascular changes during testing.

Two probes were introduced using both 2^nd^ fingers (or both 3^rd^ fingers in case of finger amputation or severe deformity). Basal pulse wave amplitude (PWA) was registered in both fingers for 5 minutes followed by sphygmomanometer cuff inflation in the 2^nd^ portion of the arm with compression of the brachial artery flow (cuff was inflated up to 200mmHg or > 60mmHg of basal systolic arterial pressure). The PWA was then recorded for another 5 minutes. Compression of arms where vascular catheters were present was avoided whenever possible. The cuff was then deflated, and a new evaluation of the PWA was performed for another 5 minutes. Each evaluation lasted between 15 - 20 minutes. An RH index (RHI), which measures vascular adaptation to a blood flow increase, was calculated automatically using Endo-PAT 2000 software (version 3.1.2) provided by the manufacturer (Figure 1).

To evaluate the normal range of the RHI in our setting, a group of 15 healthy volunteers (13 males, median age of 51 y.o., range of 31 to 61 y.o.) was selected from a cohort of kidney donors in the Nephrology Department and assessed for RH-PAT.

### Laboratory evaluation

For each admitted patient, we performed a quantification of syndecan-1, endocan, soluble E-selectin (sE-selectin) and endothelin-1 (ET-1) levels in plasma samples using a Human Premixed Multiplex kit (Magnetic Luminex® Assay, Cat. No. LXSAHM-04, kit lot No. L126978, R&D Systems®, Inc.), and analysis was performed using Luminex® 200TM xMAPTM Technology (Luminex Corp.).

Analytes concentrations were determined by laser monitoring of the spectral properties of the beads, and the amount of associated fluorescence measured was reported as median fluorescence intensity (MFI). The net MFI was calculated from the average of readings (in duplicated) for each standard and sample and subtracting the average of blank MFI. A standard five-parameter logistic (5-PL) curve fit was created for each analyte using Luminex® xPONENT® Software (version 3.1).

### Statistical analysis

Descriptive statistics included the mean and standard deviation (SD) for continuous variables and absolute and relative frequencies for categorical variables.

At baseline, for the (univariate) identification of variables significantly associated with infection, the chi-squared test was used for categorical variables, and t-test was used for continuous variables. The effects of RHI on infection were investigated using multiple logistic regression models with adjustment for other relevant variables. In accordance with sample size, only variables with a p-value from the univariate analysis less than or equal to 0.1 were considered as potential confounders. The choice of the best model was based on the likelihood ratio test for nested models or the Akaike Information Criterion (AIC).

For the models of interest, the area under the Receiver Operating Characteristic (ROC)-curve (AUC) was computed. Cut-off values for RHI were determined by maximizing the sum of sensitivity and specificity. Statistical analyses were performed with R software for statistical computation, version 2.3.3.^(33)^ The significance level was set at 0.05.

## RESULTS

During a 12-month period, 115 patients were enrolled. Twenty-nine patients were excluded. The main reasons for exclusion were unavailability to perform PAT in the first 24 hours after admission (n = 9), hemodynamic instability leading to death (n = 8), severe thrombocytopenia (n = 3), restlessness (n = 2), arterial-venous fistulae (n = 2), arterial vasospasm (n = 1) and failure to obtain an RHI measurement (n = 4). None of the patients or legal representatives refused to participate. Patient characteristics and comorbidities are shown in table 1. There was a predominance of males (67.4%) and individuals younger than 60 y.o. (59.3%).

**Table 1:** Patient characteristics and univariate analysis of septic vs control patients

	Septic (n = 58)	Controls (n = 28)	p value	Total (n = 86)
Demographics and comorbidities				
Sex - male	42 (71.4)	16 (57.1)	0.157	58 (67.4)
Age ≥ 60 years	18 (31.0)	17 (60.7)	< 0.01	35 (40.7)
Age	55.9 [1.90]	62.0 [4.20]	0.051	57.4 [1.81]
BMI	27.3 ± 5.5	27.4 ± 4.4	0.916	27.3 ± 5.1
DM2	16 (27.6)	6 (21.4)	0.540	22 (25.6)
Arterial hypertension	25 (43.1)	18 (64.3)	0.066	43 (50.0)
Dyslipidemia	22 (37.9)	13 (46.4)	0.452	35 (40.7)
Statins use	19 (32.8)	11 (39.3)	0.552	30 (34.9)
Peripheral arterial disease	12 (20.7)	9 (32.1)	0.247	21 (24.4)
COPD	10 (10.7)	3 (17.2)	0.529*	13 (15.1)
Autoimmune disease	5 (8.6)	0	0.168*	5 (4.8)
Smoking habits	20 (34.5)	8 (28.6)	0.584	28 (32.6)
Coronary disease	15 (25.9)	9 (32.1)	0.543	24 (27.9)
Hematological cancer	1 (1.7)	1 (3.6)	0.548*	2 (2.3)
HIV	9 (15.5)	3 (10.7)	0.743*	12 (14.0)
Solid neoplasia	5 (8.6)	3 (10.7)	0.712*	8 (9.3)
Clinical variables and severity scores				
APACHE score	21.1 [7.6]	19.1 [5.8)	0.194	20.5 [7.1]
SAPS 2 score	55.2 [21.1]	55.5 [17.8]	0.953	55.4 [20.0]
SOFA score	10.1 [4.4]	8.1 [3.3]	0.035	9.5 [4.2]
Shock	29 (50.0)	9 (32.1)	0.118	38 (44.2)
MAP _(mm Hg)_	75.2 [1.70]	81.0 [3.8]	0.064	76.6 [1.60]
Ionized calcium _(mmol/L)_	1.12 [0.05]	1.21 [0.03]	0.693	1.15 [0.04]
pH	7.39 [0.01]	7.42 [0.02]	0.021	7.39 [0.01]
PaCO_2__(mm Hg)_	36.9 [1.19]	40.0 [2.89]	0.628	37.6 [1.14]
Bicarbonate _(mmol/L)_	21.7 [0.56]	25.6 [1.36]	< 0.01	22.3 [0.56]
Potassium _(mEq/L)_	4.02 [0.08]	3.7 [0.96]	0.013	3.91 [0.60]
Temperature^‡^_(Celsius)_	36.8 [0.25]	36.8 [0.15]	0.905	36.8 [0.13]
Respiratory rate^‡^_(beats/minute)_	19.6 [0.77]	17.5 [1.0]	0.153	19.1 [0.64]
CRP _(mg/L)_	102.2 [12.89]	69.3 [15.4]	0.102	97.3 [13.5]
Lactate _(mmol/L)_	2.1 [2.2]	1.4 [0.5]	0.022	2.5 [2.5]
Noradrenaline _(mcg/kg/minute)_	0.25 [0.50]	0.13 [0.21]	0.105	0.38 [0.59]
Mortality _(28^th^ day)_	14 (24.1)	7 (25.0)	0.999	21 (24.4)
Biomarkers and PAT evaluation				
Ln_endocan _(pg/mL)_	7.07 ± 0.85	7.27 ± 0.58	0.403	7.11 ± 0.79
Ln_syndecan-1 _(pg/mL)_	8.61 ± 0.58	8.44 ± 0.53	0.298	8.57 ± 0.57
Ln_E-selectin _(pg/mL)_	10.79 ± 0.84	10.19 ± 0.56	0.011	10.66 ± 0.82
Endothelin-1 _(pg/mL)_	5.28 ± 3.59	5.80 ± 3.14	0.880	5.69 ± 3.47
Ln_RHI	0.53 ± 0.48	0.69 ± 0.42	0.122	0.58 ± 0.46
Diagnosis				
Neurological	Meningitis: 8 (13.7) Other: 2 (3.4)	Intracranial hemorrhage: 6 (21.4) Status epilepticus: 4 (14.3) Other: 2 (7.1)	-	22 (25.6)
Pleuropulmonary and cardiovascular	Pneumonia: 17 (29.3)	Myocardial infarction: 5	-	22 (25.6)
Intra-abdominal	Peritonitis: 5 (8.6) Pyelonephritis: 4 (6.9) Other: 2 (3.4)	GI Neoplasia: 2 (7.1) Pancreatitis: 2 (7.1) Ischemic colitis: 1 (3.6)	-	16 (27.6)
Skin and soft tissue	NF: 1 (1.7)	-	-	1 (1.2)
Systemic	Malaria: 10 (17.2) Bacteremia: 6 (10.3) Chickenpox: 2 (3.4) Tuberculosis: 1 (1.7)	Hemorrhagic shock: 3 (10.7) Polytrauma: 2 (7.1) Leukemia: 1 (3.6)	-	25 (29.0)
Confirmed microbiology	43 (74.1)	-	-	-
Positive blood cultures	24 (41.4)	-	-	-
Causative organism		-	-	-
Gram-positive bacteria	10 (17.2)			
Gram-negative bacteria	10 (17.2)			
Atypical bacteria	3 (5.2)			
Mycobacteria	2 (3.4)			
Protozoa	10 (17.2)			
Virus	4 (6.9)			
Fungus	3 (5.2)			

BMI - body mass index; DM2 - diabetes mellitus II; COPD - chronic obstructive pulmonary disease; APACHEII - Acute Physiology And Chronic Health Evaluation II; SAPS 2 - Simplified Acute Physiology Score 2; SOFA - Sequential Organ Failure Assessment; MAP - mean arterial pressure; PaCO_2_ – partial pressure of carbon dioxide; CRP - C-reactive protein; PAT - peripheral arterial tonometry; Ln - natural logarithm; NF - necrotizing fasciitis; RHI - reactive hyperemia index.

* Fischer exact test. Results expressed as n (%), mean ± standard deviation or median [interquartile range].

Within the septic group, patients were classified according to the new sepsis definition with SOFA scores ranging from 2 to 19 points. A subanalysis of the septic group revealed that 29 (50.0%) patients had septic shock.

The control group did not differ significantly from the septic group in terms of characteristics and comorbidities except for age between the two groups, with a higher prevalence of older patients in the control group (Table 1). There were no significant differences in endocan, syndecan-1 and ET-1 levels between septic and control patients, but sE-selectin levels were significantly higher in the former group (p < 0.001). Severity scores, the need for aminergic support and lactate levels were similar between groups, but the SOFA score was slightly increased in the septic group. Clinical diagnosis for each group is provided in table 1.

### Microvascular analysis - factors influencing reactive hyperemia index

The RHI was quantified in the 86 enrolled patients. Due to a skewed empirical distribution, the natural logarithm of RHI (Ln_RHI) was used. Mean Ln-RHI in the study population was 0.58 ± 0.46. Mean Ln_RHI in healthy volunteers was 0.71 ± 0.05, which was significantly increased compared with the study population (p = 0.02).

Inflammatory biomarkers (endocan, syndecan-1, sE-selectin, ET-1) were quantified in samples collected from 68 patients (53 septic and 15 controls). Endothelin-1 values in 6 (8.8%) patients evenly distributed between septic and control patients were below the lower limit of detection. Due to skewed empirical distributions, the natural logarithms of endocan, syndecan-1 and sE-selectin were used.

Univariate analysis revealed age > 60 y.o. (p = 0.023), coronary disease (p = 0.043), dyslipidemia (p = 0.015) and solid neoplasia (p < 0.001) were significantly associated with lower Ln_RHI means, higher severity scores, lower serum potassium, hypotension, the presence of shock, higher noradrenalin dose and higher lactate (Table 2). Ln_syndecan-1 was the only biomarker significantly correlated with Ln_RHI (p < 0.001, Pearson’s r -0.346). There was no significant difference in the Ln_RHI between patients with/without bacteremia, gram-positive versus gram-negative bacteria, or bacteria versus other. After adjusting for other comorbidities and severity scores through linear regression models, the same variables were significant with the exception of age, serum potassium and mean arterial pressure. Ln_sE-selectin and peripheral arterial disease also significantly influenced Ln_RHI after adjusting for other variables.

**Table 2:** Analysis of factors influencing Ln-RHI

Categorical variables (t-test for means)				
	Univariate analysis		Multivariable analysis	
	Yes	No	p value	p value; B (95%CI for OR)
Sex - male	0.59 ± 0.51	0.54 ± 0.33	0.617	NS
Age ≥ 60 y.o.	0.44 ± 0.47	0.67 ± 0.43	0.023	NS
DM2	0.49 ± 0.42	0.61 ± 0.48	0.347	NS
Arterial hypertension	0.50 ± 0.46	0.65 ± 0.45	0.127	NS
Dyslipidemia	0.44 ± 0.47	0.68 ± 0.43	0.015	0.03; -0.908 (-1.463; -0.320)
Statins use	0.43 ± 0.47	0.65 ± 0.43	0.031	0.029; 0.547 (0.058; 1.035)
Peripheral arterial disease	0.58 ± 0.47	0.57 ± 0.44	0.939	< 0.01; 0.490 (0.220; 0.880)
COPD	0.47 ± 0.52	0.60 ± 0.45	0.351	NS
Autoimmune disease	0.60 ± 0.68	0.57 ± 0.45	0.911	NS
Smoking habits	0.55 ± 0.56	0.59 ± 0.41	0.709	NS
Coronary disease	0.42 ± 0.46	0.64 ± 0.45	0.047	0.02; -0.304 (-0.590; -0.040)
Hematological cancer	1.06 ± 0.39	0.56 ± 0.45	0.135	NS
HIV	0.61 ± 0.43	0.57 ± 0.46	0.775	NS
Metastatic solid neoplasia	0.07 ± 0.51	0.63 ± 0.42	< 0.01	< 0.01; -0.275 (-0.72; -0.11)
Shock	0.31 ± 0.41	0.79 ± 0.39	< 0.01	< 0.01; -0.261 (-0.53; -0.07)
**Continuous variables (Pearson’s correlation)**				
** **	**Univariate analysis**			**Multivariable analysis**
** **	**Pearson’s r**		**p value**	**p value; B (95%CI for the OR)**
APACHE II score	-0.406		< 0.01	< 0.01; -0.362 (-0.039 to -0.010)
SAPS 2 score	-0.359		< 0.01	< 0.01; -0.320 (-0.013 to -0.003)
SOFA score	-0.314		< 0.01	< 0.01; -0.414 (-0.067 to -0.022)
MAP _(mmHg)_	0.341		0.001	NS
Ionized calcium _(mmol/L)_	0.201		0.540	NS
pH	0.206		0.067	NS
pCO_2__(mm Hg)_	-0.140		0.895	NS
Potassium _(mEq/L)_	-0.224		0.023	NS
Temperature _(Celsius)_	0.071		0.546	NS
Respiratory rate _(beats/minute)_	-0.102		0.556	NS
CRP _(mg/L)_	-0.135		0.445	NS
Lactate _(mmol/L)_	-0.499		< 0.01	0.01; 0.410 (-0.117 to 0.936)
Noradrenaline _(mcg/kg/minute)_	-0.348		< 0.01	NS
Ln_endocan _(pg/mL)_	-0.137		0.265	NS
Ln_syndecan-1 _(pg/mL)_	-0.346		< 0.01	0.010; -0.276 (-0.483 to -0.069)
Ln_E-selectin _(pg/mL)_	-0.005		0.965	0.024; -0.245 (-0.273 to -0.02)
Endothelin-1 _(pg/mL)_	0.027		0.832	NS

APACHE II - Acute Physiology and Chronic Health Evaluation II; B - coefficient; BMI - body mass index; COPD - chronic obstructive pulmonary disease; CRP - C-reactive protein; DM2 - Diabetes Mellitus II; Ln - natural logarithm; PAT - peripheral arterial tonometry; OR - odds ratio; RHI - reactive hyperemia index; SAPS II - Simplified Acute Physiology Score II; SOFA - Sequential Organ Failure Assessment.

### Microvascular analysis - association of reactive hyperemia index and infection

In the comparison of Ln_RHI between septic and control groups, those with infection had a lower average (0.53 ± 0.48 versus 0.69 ± 0.42, respectively); however, the difference was not statistically significant (p = 0.122). To evaluate further interactions between patient characteristics and the presence of sepsis, a multivariable analysis adjusting the effect of Ln_RHI for significant and marginally significant variables from the univariate analysis was performed. The best regression model consisted of Ln_RHI (p = 0.024) and age (p = 0.002) as explanatory variables (Model A - Table 3); both variables are negatively associated with sepsis. The model predicts that within each age group, each 0.1 decrease in Ln_RHI, increases the odds for sepsis by 14.6% (1/e^-1.368*0.1^ = 1.146). For any fixed value of RHI, individuals 60 y.o. or older have an 80.4% reduced risk for sepsis than those younger than 60 y.o. The interaction between age group and Ln_RHI was not significant (p = 0.821); thus, the association between Ln_RHI and sepsis does not significantly vary between the two age groups. No other variables (including hypertension and severity markers) significantly improved the model.

**Table 3:** Estimates from the logistic regression models studying the effect of Ln-RHI/Ln-sE-selectin and age group on the probability of infection

	Variable	Coefficient	Standard error	p value	OR	95% CI for OR	ROC AUC; p value	Se/Sp	PPV/NPV	Threshold for predicted probabilities (Se/Sp)
**Model A**	Ln_RHI	-1.368	0.605	0.024	0.255	0.078 - 0.833	0.726; p < 0 .01	75%/71%	75%/71%	0.55 (86%/54%)
	Age									
	< 60 y.o.	Reference	-	-	-	-				
	≥ 60 y.o.	-1.629	0.538	0.002	0.196	0.068 - 0.563				
**Model B**	Ln_sE-selectin	1.308	0.504	0.009	3.698	1.398 - 9.931	0.780; p < 0.01	94%/20%	81%/50%	0.52 (59%/93%)
	Age									
	< 60 y.o.	Reference	-	-	-	-				
	≥ 60 y.o.	-1.412	0.669	0.035	0.244	0.066 - 0.904				

AUC - area under curve; CI - confidence interval; Ln - natural logarithm; OR - odds ratio; PPV - positive predictive value; NPV - negative predictive value; RHI - reactive hyperemia index; ROC - receiver operator curve; Se - sensitivity; Sp - specificity.

Alternatively, we performed the same analysis considering Ln_sE-selectin as a predictor of sepsis based on the strong association with the septic group found in the univariate evaluation (Model B - Table 3). The best model consisted of age (p = 0.035) and sE-selectin (p = 0.02) as explanatory variables. In each age group, the model predicted that an increase of 0.1 in Ln_sE-selectin values increases the odds for sepsis by 13.9%. Individuals 60 y.o. or older exhibit 75.6% lower odds for sepsis than those younger than 60 y.o. The interaction between the age group and Ln_sE-selectin was not statistically significant (p = 0.538).

Receiver operating characteristic curves and corresponding values of the Area Under Curve (AUC), sensitivities, specificities and positive (PPV) and negative (NPV) predictive values for the regression models were computed. In Model A, the best Ln_RHI cut-off values were 1.43 (RHI 4.2) for patients younger than 60 y.o. and 0.41 (RHI 1.5) for patients 60 y.o. or older. In Model B, the best Ln_sE-selectin cut-off values were 9.5 (sE-selectin: 12,708pg/mL) and 10.5 (sE-selectin: 37,421pg/mL) for patients younger than 60 y.o. and patients 60 y.o. or older, respectively.

## DISCUSSION

The present study aimed to clarify the role of RH-PAT in sepsis by comparing a group of septic patients in the ICU with a group of critically ill patients without evidence of infection. After adjusting for variables known to influence RHI, we found a significant difference influenced by age between patients with and without sepsis.^(34-37)^ The RHI was negatively associated with sepsis. Thus, the lower RHI, the higher the risk of the patient being septic. This association was independent of disease severity, comorbidities and other factors known to influence the RHI. This finding suggests that although disease severity and comorbidities are the main factors influencing RHI, the presence of infection (irrespective of the state of sepsis or septic shock) seems to decrease the RHI to a greater extent than that in other critical illnesses.

Age highly influenced the predictive values of RHI. For the same RHI, the odds for infection were significantly lower in older patients. Reactive hyperemia index cut-off values also varied widely between the two age groups. Despite good threshold sensitivity (86%), the lack of specificity makes these values clinically unreliable. Endothelial frailty may be a justification for this difference as older patients may already have a dysfunctional endothelium with a lower basal RHI due to age and comorbidities. Therefore, in older patients, the presence of infection may be sufficient to decrease endothelial function, and a lower RHI threshold may be reasonable to aid in the diagnosis of infection. In contrast, younger patients may have a protective functional endothelium; thus, a severe infection with a deep systemic inflammatory response would be required for an effect as intense as that seen in older patients.

Syndecan-1 is a transmembrane heparan sulfate proteoglycan, and syndecan-1 serum levels increase in the setting of glycocalyx degradation.^(38,39)^ There are a few reports of increased serum syndecan-1 levels in septic patients.^(40-43)^ Glycocalyx integrity is essential for the shear stress sensing mechanism that leads to the NO-dependent vasodilatory response observed in reactive hyperemia, and the absence of syndecan-1 significantly decreases endothelial nitric oxide synthetase (eNOS) and vascular reactivity.^(44,45)^ As syndecan-1 levels increase in serum due to sepsis-induced cleavage, the reactive hyperemia response is hypothesized to be impaired, and RHI would decrease. E-selectin is a glycan binding protein that is typically absent in the endothelial wall and is upregulated and mobilized by vascular endothelial cells to the cell surface facing the blood vessel lumen in response to inflammatory mediators (bacterial lipopolysaccharide, interleukin - IL-1β, and tumor necrosis factor-alpha - TNF-α). Its main function is to initiate neutrophil extravasation into tissues in acute and chronic inflammatory diseases.^(46,47)^ Soluble E-selectin becomes available in plasma upon apoptosis of activated endothelial cell, correlating with the degree of endothelial activation, and higher plasma levels are associated with sepsis and sepsis severity.^(29,48,49)^ Together, the independent association of RH-PAT with syndecan-1 and sE-selectin levels in our results suggests that glycocalyx integrity and endothelial activation may have a role in vascular reactivity, more specifically with reactive hyperemia.

Despite the promising results of syndecan-1 and sE-selectin, endocan, a proteoglycan secreted by the vascular endothelium in septic patients, was not associated with RH-PAT or sepsis.^(50-53)^ As pneumonia was underrepresented in our study population and endocan is mainly produced by the kidney and lung in the presence of inflammatory mediators, our results may have been underpowered, and such an association was not identified. Endothelin-1 (ET-1) is an endothelial derived factor that regulates vascular tone and remodeling, induces adhesion molecules in the endothelial wall and adherence of polymorphonuclear cells, and leads to oxidative stress with endothelial damage.^(54,55)^ Although ET-1 levels are usually increased in cardiovascular diseases and sepsis, we did not find such an association or any association with RH-PAT. Possible explanations include that ET-1 levels may not differ in a population with high comorbidities and critical disease even in the presence of infection. Alternatively, as plasma ET-1 levels are 100-fold lower that on a the vascular wall, plasma quantifications may not be sensitive enough.^(56)^

Our data confirmed our hypothesis that when measured by PAT, sepsis is associated with an impaired reactive hyperemia response to a greater degree than with other nonseptic conditions. This finding has not been previously reported using this method. These results also clarified the association between markers of endothelial activation and glycocalyx degradation and the reactive hyperemia response, which has not been previously explored in other studies. However, although a definite trend is evident, our model using RHI has only moderate sensitivity and specificity, which may be explained by the fact that RHI is affected by numerous variables as evidenced in our initial analysis.

Additional work will be required to fully understand the role of RH-PAT in sepsis as there are some limitations in our study. Given the lack of a gold standard for the diagnosis of sepsis, the definition used in our study may have failed to correctly classify patients. A larger sample size would be required to attenuate the effect of variables influencing RH-PAT, but the costs associated with each PAT measurement make this difficult in research and in clinical practice. A multicentered study including other ICUs is necessary to decrease potential selection bias. Although this ICU is part of the infectious diseases department, it is considered a general ICU and receives adult patients with any type of critical condition. Early mortality in the ICU is high, and RH-PAT requires hemodynamic stability to be achieved. Unfortunately, a substantial number of patients died before recruitment, which could have skewed the results. The test was also performed after the start of antimicrobials and inotropes, which directly impact arterial tone and potentially altered the response to the test.

Nonetheless, although RH-PAT does not at present seem to be of clinical value for distinguishing septic patients from other patients admitted to the ICU, there was a strong association with every measured marker of disease severity, suggesting that RH-PAT may be useful for accessing prognosis. However, except for septic and cardiogenic shocks, where RH-PAT has been proven to be impaired, studies correlating RH-PAT and other conditions in the ICU are lacking.^(57-59)^ Furthermore, sepsis is currently defined as organic dysfunction in the presence of infection, and the latter is responsible for the inflammatory cascade that leads to organic malfunction in some patients, distinguishing the normal inflammatory response to infection from sepsis. If RH-PAT is proven to be correlated with organic dysfunction, it may become a useful method to distinguish patients with simple infections from patients with microvascular dysfunction and progression to sepsis.

As RH-PAT is noninvasive and has almost no contraindications or complications, it is a desirable method for evaluating reactive hyperemia. However, future studies should focus on the prognostic value of the test.

## CONCLUSION

Reactive hyperemia-peripheral arterial tonometry seems to be impaired in septic patients compared with patients with noninfectious critical illness. However, reactive hyperemia-peripheral arterial tonometry is highly influenced by age and lacks specificity, which may render it clinically unreliable to distinguish septic patients from nonseptic patients. Soluble E-selectin and syndecan-1 were independently associated with reactive hyperemia-peripheral arterial tonometry, which may suggest common pathological mechanisms. Our results revealed a strong association between reactive hyperemia index and disease severity, and further studies should clarify whether reactive hyperemia-peripheral arterial tonometry can be used as a prognostic indicator in patients admitted to the intensive care unit.
